# Drift and Genome Complexity Revisited

**DOI:** 10.1371/journal.pgen.1002092

**Published:** 2011-06-09

**Authors:** Kenneth D. Whitney, Bastien Boussau, Eric J. Baack, Theodore Garland

**Affiliations:** 1 Department of Ecology and Evolutionary Biology, Rice University, Houston, Texas, United States of America; 2 Department of Integrative Biology, University of California Berkeley, Berkeley, California, United States of America; 3 Laboratoire de Biométrie et Biologie Evolutive, Université de Lyon, Université Lyon 1, CNRS, UMR5558, Villeurbanne, France; 4 Department of Biology, Luther College, Decorah, Iowa, United States of America; 5 Department of Biology, University of California Riverside, Riverside, California, United States of America; Yale University, United States of America

## Introduction

Recently, Whitney and Garland [1] (hereafter “WG”) reanalyzed a dataset presented in Lynch and Conery [2] (hereafter “LC”) using phylogenetic statistical techniques. Contrary to LC, WG found little support for the idea that *N_e_u* (the product of effective population size and the mutation rate) is statistically related to genome size or six other genomic attributes. Lynch [3] has responded with criticisms of the WG approach and interpretations. Below we carefully consider these criticisms, present additional analyses, and conclude that the WG analyses are robust. In addition, we explore the consistency of some predictions of the mutational-hazard (MH) hypothesis [3] and provide some guidance regarding future tests.

Given that both analyses used the same dataset, the heart of the issue is the choice of analysis techniques and interpretation of results. Below, we use the terms “phylogenetic” and “nonphylogenetic” to describe the techniques employed by WG and LC, respectively. “Nonphylogenetic” remains in quotes because, in fact, species-level regression or correlation analyses that do not explicitly incorporate phylogenetic history do assume a particular phylogeny—a star phylogeny (polytomy) in which all species are equally related and all branches have equal lengths [4], [5] .

## The Appropriateness of Phylogenetic Analyses

Lynch [3] argues that both *N_e_u* and measures of genome complexity (e.g., genome size) are so evolutionarily labile that analyses incorporating a hierarchical phylogenetic tree are unnecessary and potentially misleading (but see [6]). The issue can be empirically addressed [7], [8]. The key test of whether a phylogenetic or “nonphylogenetic” regression analysis is more appropriate examines the regression residuals for phylogenetic signal [8], [9]. Phylogenetic signal in the residuals is evidence that the evolutionary response of the dependent variable to the independent variable was not so rapid as to make phylogeny unimportant in regression analyses. This was the agnostic approach taken in WG, letting the statistics indicate the best-fit model. The phylogenetic models had better fit (see Table 1 in [1]), indicating significant phylogenetic signal in the residuals. These models did not support the hypothesis that *N_e_u* explains a significant fraction of the variation in genomic attributes such as genome size.

**Table 1:** Univariate measures of phylogenetic signal for log_10_-transformed traits in the dataset.

Trait	K	*P*
*N_e_u*	0.93	<0.001
Genome size (Mb)	1.25	<0.001
Gene number	1.43	<0.001
Half-life of gene duplicates	0.62	0.047
Intron size	0.72	0.002
Intron number	1.47	0.045
Transposons (number)	0.63	0.002
Transposons (fraction of genome)	1.08	<0.001

K varies from 0 to 1 to >1, indicating, respectively, no phylogenetic signal, that relatives resemble each other as much as expected under Brownian motion–like evolution, and that relatives are more similar to each other than expected under Brownian motion [7]. *P*-values indicate significant phylogenetic signal based on randomization tests of the mean squared error. Results are from the Picante package in R [34], [35] utilizing the phylogeny presented in [1] with all = 1 branch lengths.

Although the key insight regarding trait lability is determined from the phylogenetic signal of the regression residuals, it can also be instructive to examine phylogenetic signal for particular traits. Table 1 presents estimates of phylogenetic signal (K) for the dataset under discussion; all traits show significant (and often extremely strong) phylogenetic signal, indicating that species cannot be considered statistically independent entities for any of these traits [7]. Such strong phylogenetic signal may be counterintuitive for *N_e_u,* which is a population-level trait as opposed to a “standard” individual-level morphological trait. However, *N_e_* can be construed as an emergent trait that reflects several other traits (e.g., mating system, dispersal ability, social group size, body size) that generally do show phylogenetic signal (e.g., [7]). In any case, the empirical data do not support Lynch's contention that *N_e_u* (as estimated by π_s,_ the average nucleotide heterozygosity at silent sites) is so labile as to “hav[e] no shared phylogenetic history” across the species in the dataset.

Next, Lynch argues that phylogenetic techniques are inappropriate for the current dataset because “. . . phylogenetic inertia is overshadowed by other evolutionary effects. For example, for the two most closely related species . . . mouse and human . . . numerous shared features of genome architecture are a consequence of convergent evolution, not shared ancestry.” He observes that genome sizes in different species may be determined by the abundances of different transposable element (TE) families. Although it is certainly true that genome architecture can be superficially similar because of convergent evolution, and that such convergence can evolve via different underlying components (e.g., different TEs in the case of genome size), these observations do not automatically override the necessity for phylogenetic analyses. Phylogenetic nonindependence must be accounted for if it exists, no matter how it arises. Phylogenetic signal in the residuals of the regression of genome size on *N_e_u* (see WG and Table 2 of the current article) indicates that related species could share similar values of other traits (aside from *N_e_u*) that influence genome size. We posit that traits influencing the proliferation of TEs (e.g., mating system, methylation propensity, RNAi-mediated interference) show phylogenetic signal and are partly responsible for the nonindependence observed among residual genome sizes of closely related species. Another non-mutually-exclusive hypothesis is that related taxa share physiological traits that partly determine the environments in which they can live (e.g., [10], [11]), and that the resulting shared environmental conditions have caused selection favoring similar-sized genomes. Regardless of one's ability to identify the lower-level traits involved, phylogenetic nonindependence of residuals is present in the current dataset (WG and Table 2 of the current article), and ignoring it can lead to incorrect inferences about associations between traits.

**Table 2:** Relationships between *N_e_u* and genome size as estimated by three types of linear regression models: “nonphylogenetic” (OLS), phylogenetic generalized least squares (PGLS; equivalent to phylogenetically independent contrasts), and phylogenetic regression in which the residual variation is modeled as an Ornstein-Uhlenbeck process (RegOU).

Model	Topology	Branch Lengths	ln Max Likelihood	*N*	*b*	*r* ^2^	*d*	*P* for Regression
***Ordinary Least Squares (OLS)***								
	--	--	−25.53	29	−1.17	0.64^a^		<0.001
***Phylogenetic Generalized Least Squares (PGLS)***								
	Coelomata	All = 1	−23.51	29	−0.33	0.08		0.137
	Coelomata	Fossil	−32.36	29	−0.25	0.04		0.326
	Coelomata	rRNA	−39.76	29	0.05	0.00		0.983
	Ecdysozoa	All = 1	−24.06	29	−0.34	0.09		0.124
	Ecdysozoa	Fossil	−32.33	29	−0.26	0.04		0.313
	Ecdysozoa	rRNA	−39.73	29	0.00	0.00		0.994
***Phylogenetic Regression under an Ornstein-Uhlenbeck Process (RegOU)***								
	Coelomata	All = 1	−22.59*	29	−0.20	0.04	1.31	0.328
	Coelomata	Fossil	−25.51	29	−1.14	0.61	0.00	<0.001
	Coelomata	rRNA	−25.55	29	−1.16	0.64	0.00	<0.001
	Ecdysozoa	All = 1	−23.08*	29	−0.20	0.04	1.35	0.332
	Ecdysozoa	Fossil	−25.51	29	−1.14	0.61	0.00	<0.001
	Ecdysozoa	rRNA	−25.55	29	−1.16	0.64	0.00	<0.001

For each of the phylogenetic regression models, two alternate tree topologies were used, each with three alternate sets of branch lengths. Log_10_ (genome size) was regressed on log_10_(*N_e_u*); *b* = regression slope; *d* = REML estimate of the OU parameter. PGLS and OLS models are compared using ln maximum likelihoods, with a higher likelihood taken as evidence of a better-fitting model. OU and OLS models are compared with ln maximum likelihood ratio tests; asterisks (*) indicate that the OU model fit significantly better than OLS (*P*<0.05). All analyses were done using the Regressionv2.m Matlab program of [9], available from TG on request. Methods and full results are available at http://hdl.handle.net/1911/61373.

a Lynch and Conery [2] reported *r* ^2^ = 0.66; the discrepancy apparently arises because their analysis used 30 species, only 29 of which were reported in their online supplement.

Finally, Lynch makes two general criticisms of phylogenetic methods. First, he asserts “it can be shown” that the phylogenetically independent contrast method inflates the sampling variance of the independent variable and decreases *r* ^2^ values by ≈30%. No justification or citation is given for this assertion, and we know of no such bias. Moreover, *r* ^2^ values are generally not directly comparable across “nonphylogenetic” and phylogenetic regression models [9]. Second, citing [12], Lynch states that ordinary least-squares (OLS) correlations are “on average, unbiased” and that similar correlations are expected “whether or not shared phylogenetic history is accounted for.” Indeed, empirically, parameter estimates from the two types of analyses are often similar (see also [5], [13]). However, this *average* outcome across studies does not prevent phylogenetic versus “nonphylogenetic” analyses from giving very different answers for a particular dataset, which is clearly the case here. Thus, any conclusion that a “nonphylogenetic” analysis will always provide the correct inference is not warranted.

## Estimation of *N_e_u*

Lynch identifies three issues relating to *N_e_u* and to estimating *N_e_u* via π_s_ : 1) estimates of π_s_ are associated with high sampling variance; 2) because of constraints on *N_e_* and *u*, many prokaryote species will have similar *N_e_u* values; and 3) π_s_ in unicellular species is subject to downward bias resulting from selection on silent sites, perhaps causing prokaryotic *N_e_u* estimates to be off by more than an order of magnitude. These issues are properly viewed as criticisms of the dataset itself, not the chosen analysis. They are equally applicable to the OLS analysis of LC and have no bearing on whether a phylogenetic versus “nonphylogenetic” analysis is more appropriate.

We note that error in the independent variable can be incorporated into both phylogenetic and “nonphylogenetic” regression analyses using special techniques (e.g., [14]). However, such techniques require that the error be quantified. For the current dataset, error in π_s_ is not quantified, and thus neither we nor Lynch have the opportunity to apply such techniques.

## Tree Topologies and Branch Lengths

Lynch argues that potential uncertainties associated with tree topology and branch lengths weaken the conclusions of WG. We agree that errors in topologies and branch lengths can influence the outcomes of phylogenetically based statistical analyses [4], [5], [15]. However, the key point is that a “nonphylogenetic” analysis (e.g., the OLS regression performed in LC) is not phylogeny-free. Regression analyses assume that residuals in the dependent (Y) variable are independent and identically distributed. Under Brownian-motion-like evolution, the only phylogenetic tree that generates the appropriate variance–covariance matrix (an identity matrix) is a star phylogeny, in which each taxon is equally related to all other taxa and branch lengths are equal [4], [5]. In effect, the LC analysis assumes that humans are no more closely related to mice than to bacteria. Clearly, if there are critical errors in tree topology (and branch lengths) that undermine the conclusions of the alternate analyses under discussion here, then they are found in the star phylogeny assumed by LC.

The sensitivity of a phylogenetic comparative analysis is often assessed by examining alternative topologies and/or branch lengths (e.g., [16]). To assess the robustness of the WG results, we have investigated a second topology suggested by Lynch [3] and two additional sets of branch lengths. The WG topology followed the “Coelomata hypothesis,” whereas the alternate topology reflects the “Ecdysozoa hypothesis” and unites nematodes and arthropods in a monophyletic group [17]. We did not investigate a third topology suggested by Lynch, as it is not supported in recent analyses [18]–[20]. Three sets of branch lengths were calculated for the two trees: arbitrary lengths (all = 1) as in WG, lengths derived from fossil-based divergence times, and lengths based on ribosomal RNA substitutions. Full methodological details are available as supplementary material from the Rice Digital Scholarship Archive at http://hdl.handle.net/1911/61373. Consistent with the WG results, none of the six phylogenetic generalized least-squares (PGLS) analyses found statistically significant relationships between *N_e_u* and genome size, and the models using all = 1 branch lengths best fit the data (had the highest likelihoods) regardless of the topology (Table 2). Thus, the conclusion of no relationship between *N_e_u* and genome size appears robust to substantial variation in topologies and branch lengths.

The analyses of topologies and branch lengths described above (including the star topology assumed by OLS) all assume a Brownian motion–like model of residual trait evolution. If residual evolution has not been Brownian motion–like, then both PGLS and OLS analyses may be suspect. This is why WG explored an additional model—the Ornstein-Uhlenbeck (OU) model, which is based on a diffusion process in which a particle wanders via a random walk, but is bounded by a restraining force whose power increases with distance from the starting point [7], [21]. Felsenstein ([21], p. 464) argued that the OU process is a good model for “the motion of a population which is wandering back and forth on a selective peak under the influence of genetic drift” or for “the wanderings of an adaptive peak in the phenotype space.” WG verified that a regression model with residuals modeled as an OU process (RegOU; [9]) fit significantly better than OLS, and found that it also did not support a relationship between *N_e_u* and genome size. We have expanded those results by examining RegOU models for the full set of topologies and branch lengths (Table 2). Again, the best-fitting models for both topologies had starter branch lengths of 1.0 and did not support a significant relationship between *N_e_u* and genome size (Table 2).

## Thresholds

Lynch [3] states that the MH hypothesis predicts threshold (nonlinear) relationships on a log scale between *N_e_u* and measures of genome complexity, including genome size. Therefore, he argues that the WG analyses of linear relationships are inherently flawed. We find this argument inconsistent, given that a central analysis of LC examines the relationship between log *N_e_u* and log genome size and reports a highly significant *linear* relationship (*r* ^2^ = 0.66; their Figure 1b). Furthermore, neither LC nor [22] discuss thresholds or nonlinearity in the *N_e_u* / genome size relationship, nor is there obvious visual evidence of thresholds in the data (Figure 1b of [2]; Figure 4.8 of [22]; Figure 3a of [1]). As with genome size, three of the remaining six attributes analyzed in WG (gene number, the half-life of gene duplicates, and intron size) are clearly not associated with thresholds in LC, given that they are presented as linear relationships or, in the case of gene number, a slightly curvilinear relationship (see Figures 1–3 of [2]).

WG did perhaps err in conducting linear analyses of *N_e_u* against three other genomic attributes associated with thresholds in LC: intron number, transposon number, and transposon fraction. However, Lynch's argument that a “substantial reduction in the correlation of [*N_e_u* with] genomic attributes” does not contradict the MH hypothesis but instead follows from WG's use of phylogenetic techniques is not correct: the problem is not that WG used PGLS, but that within PGLS, they chose to model linear rather than threshold relationships for these particular attributes. PGLS is capable of modeling any relationship possible with OLS [23], including linear, polynomial, and break-point relationships (e.g., segmented regression [24]).

A simple approach to test for threshold effects of *N_e_u* is via the PGLS equivalent of ANCOVA [9] on two groups separated into low versus high *N_e_u*. Of the 15 species with *N_e_u* and intron number data in the LC dataset, only two fall into the “high” *N_e_u* class (*N_e_u*>0.015); similarly, of the 18 species with transposon number (or fraction) data, only three fall into the “high” *N_e_u* class (*N_e_u*>0.0128). These highly unbalanced designs do not allow confidence in analysis via either regular or phylogenetic ANCOVA. Therefore, the LC dataset does not permit robust conclusions about the responses of introns and transposons to *N_e_u* thresholds, regardless of whether one utilizes phylogenetic or “nonphylogenetic” techniques.

## Lessons from Other Studies

Lynch takes issue with WG's interpretations of two other studies. In both cases, he argues that the metric used to estimate the strength of drift/selection (allozyme-derived *N_e_* [25]; *K_a_/K_s_* [26]) is inappropriate for investigating relationships between drift and genome complexity. We argue below that allozyme-derived *N_e_* is in fact informative for the dataset in [25]. The merits of *K_a_/K_s_* have been discussed elsewhere [26]–[28] and will not be treated further here. Despite concerns about the *K_a_/K_s_* metric, Lynch [3] nonetheless views the results in bacteria [26] as “compelling support” for the MH hypothesis.

Whitney et al. [25] examined allozyme-based estimates of *N_e_* and genome size for 205 species of seed plants; using phylogenetically independent contrasts, no significant relationship was detected. (OLS analysis found a significant negative relationship, apparently the basis of Lynch's characterization of the results as “consistent” with the MH hypothesis.) Lynch argues first that allozyme data are not useful for estimating *N_e_u*, because allozymes are products of protein-sequence variation and thus are less reliable surrogates of neutral variation than silent sites. We agree that there are likely constraints on allozyme *H* that limit the maximum *N_e_u* that can be estimated; however, it does not follow that the signal of *N_e_u* is completely erased. In fact, as discussed in [25], a significant positive correlation exists between allozyme-based and sequence-based *N_e_u* estimates in a subset of the plant dataset. Furthermore, for a subset of the LC dataset for which allozyme data were available, allozyme-based *N_e_u* was as strongly related to genome size as was sequence-based *N_e_u* [25]. Lynch also argues that regressions in [25] should have used *N_e_u* rather than *N_e_*. In that analysis, *N_e_* was calculated from heterozygosity *H* via *N_e_* = ((1–*H*)^−2^–1)*/*(8*u*), assuming a constant *u* of 10^−5^. That assumption means that, computationally, it makes absolutely no difference whether *N_e_u* or *N_e_* were used; neither had a significant relationship with genome size in phylogenetic analyses.

Kuo et al. [26] analyzed 42 paired bacterial genomes, using the efficacy of purifying selection in coding regions (as estimated by *K_a_/K_s_*) to quantify genetic drift. Bacterial taxa experiencing greater levels of genetic drift—implying a smaller evolutionary *N_e_*—had smaller genomes. Lynch [3] argues that these results support the MH hypothesis because “the theory predicts that with increasing power of random genetic drift, effectively neutral genomic features will evolve in the direction of mutation bias” and because “there is a deletion bias in bacteria” in contrast to an insertion bias in eukaryotes. Thus, the predicted *N_e_u* and genome size/complexity relationship is positive for prokaryotes and negative for eukaryotes. These statements appear to represent a revision of the MH hypothesis, which in previous treatments [2], [22] had assumed an insertion bias in both groups and a continuous, negative *N_e_u* versus genome size relationship across prokaryotes and eukaryotes.

The assertion that mutation bias differs in direction for prokaryotes and eukaryotes is difficult to evaluate. We note that studies examining mutation bias typically find a deletion bias in both groups (e.g., [29] and references therein). More importantly, most of these studies use sequence data from diverged lineages to estimate the ratio of insertions to deletions. In previous discussions, Lynch has argued [22], [30] that such studies do not accurately estimate the quantity of interest (de novo mutation bias), in contrast to lab mutation accumulation studies involving relaxation of selection. We agree: indels in sequence data from naturally diverged lineages reflect not only mutation but also subsequent selection and drift and thus may not represent the de novo mutation spectrum. However, lab mutation accumulation studies [31], [32] are simply too few to allow generalizations about mutation biases in prokaryotes versus eukaryotes. The lack of hard data on de novo mutation bias means that any nonzero correlation between *N_e_u* and genome size can be judged “consistent” with the MH hypothesis simply by claiming the appropriate mutation bias.

Regardless, the new prediction for decreasing prokaryotic genome size with decreasing *N_e_u* is not supported by the LC dataset, whether analyzed using “nonphylogenetic” or phylogenetic methods. We regressed genome size on *N_e_u* using both OLS and PGLS for just the seven bacterial species and found no statistical relationship in either analysis (*b* = −0.19 and −0.11, *P* = 0.47 and 0.49, respectively). Although the sample size is small, we note the trends are for genome size and *N_e_u* to move in opposite directions, counter to the prediction if a deletion bias in bacteria is assumed.

In summary, the datasets of Whitney et al. [25] and of LC do not support the MH hypothesis regardless of the assumed direction of mutation bias. The Kuo et al. data [26] contradict the MH hypothesis, assuming a universal insertion bias, but support it under an assumption of a deletion bias in prokaryotes. We conclude, as did WG, that current comparative datasets examining drift and genome size provide little support for the MH hypothesis.

## Conclusions

We agree with Lynch [3] that the MH hypothesis should not be rejected based on the difficulty of performing formal hypothesis tests. We note, however, that such difficulty does not in turn justify acceptance based on inappropriate statistical models. We find the theoretical population genetic basis of the original LC argument sound: smaller effective population size should result in an increasing role for drift relative to selection and an increasing probability of fixation of slightly deleterious mutations that alter genome size and complexity. Our focus, however, is not whether effective population size plays a role, but how important it might be relative to numerous other factors that might influence genome size and complexity. Does *N_e_u* explain 66% of the variation in genome size across the tree of life, 6%, or 0.6%? The WG analysis and those presented herein suggest that, given the demonstrated phylogenetic nonindependence of the data at hand, the 66% estimate claimed by LC is far too high; in fact, any influence of *N_e_u* on genome size is not statistically detectable in better-fitting phylogenetic regression models (Table 2). Finally, we question whether simple regression models (regardless of whether they are phylogenetic or “nonphylogenetic”) can ever provide unequivocal support for the MH hypothesis. One of the major criticisms expressed in WG and in [33] is that *N_e_u* is highly correlated with other aspects of organismal biology, including body size, mating system, developmental rate, and metabolic rate. Thus, comparative analyses using only *N_e_u* as a predictor variable may be uninformative about the actual mechanisms driving genome size and complexity; multivariate analyses are needed.
